# Combined Effect of Anticancer Agents and Cytochrome C Decorated Hybrid Nanoparticles for Liver Cancer Therapy

**DOI:** 10.3390/pharmaceutics10020048

**Published:** 2018-04-12

**Authors:** Wejdan Al-Shakarchi, Ali Alsuraifi, Mohammed Abed, Marwan Abdullah, Alan Richardson, Anthony Curtis, Clare Hoskins

**Affiliations:** 1 Institute of Science and Technology in Medicine, School of Pharmacy, Keele University, Keele ST5 5BG, UK; w.n.s.al-shakarchi@keele.ac.uk (W.A.-S.); a.t.y.alsuraifi@keele.ac.uk (A.A); m.n.abo.donia@keele.ac.uk (M.A.); m.i.abdullah@keele.ac.uk (M.A.); a.richardson1@keele.ac.uk (A.R.); a.d.m.curtis@keele.ac.uk (A.C.); 2 College of Pharmacy, Mosul University, Mosul 41002, Iraq; 3 College of Dentistry, University of Basrah, Basrah 61004, Iraq

**Keywords:** apoptosis, liver cancer, hybrid nanoparticle, cytochrome C, combination therapy

## Abstract

Hepatocellular carcinoma is an aggressive form of liver cancer that displays minimal symptoms until its late stages. Unfortunately, patient prognosis still remains poor with only 10% of patients surviving more than five years after diagnosis. Current chemotherapies alone are not offering efficient treatment, hence alternative therapeutic approaches are urgently required. In this work, we highlight the potential of combination of treatment of hepatocellular carcinoma with existing chemotherapies in combination with pro-apoptotic factor cytochrome C. In order to allow cytochrome C to cross the cellular membrane and become internalized, it has been immobilised onto the surface of hybrid iron oxide-gold nanoparticles. This novel approach has been tested in vitro on HepG2, Huh-7D and SK-hep-1 cell lines in order to elucidate potential as a possible alternative therapy with greater efficacy. The data from our studies show consistently that combining treatment of clinically used anticancer agents (doxorubicin, paclitaxel, oxaliplatin, vinblastine and vincristine) significantly increases the levels of apoptosis within the cell lines, which leads to cellular death. Hence, this combined approach may hold promise for future treatment regimes.

## 1. Introduction

Hepatocellular carcinoma (HCC) is the most common type of primary liver cancer accounting for approximately 75% of all cases globally [1]. It often occurs in patients with chronic liver diseases, such as cirrhosis caused by hepatitis B or hepatitis C infection [2]. HCC commonly remains undiagnosed until its late stages due to its lack of specific symptoms, and these include: loss of appetite, vomiting, stomach ache, itchy or jaundiced skin [3]. In the UK, only 10% of patients survive five years after diagnosis [4]. Current chemotherapeutic regimes use doxorubicin and cisplatin [5]. However, these drugs possess low efficacy due to their poor physicochemical properties resulting in rapid clearance and inability to permeate the tumour site. Therefore, more effective therapies or formulation strategies are required in order to improve patient outcomes.

One such strategy being employed is the exploitation of the apoptosis pathway. Apoptosis, or programmed cell death, is one of the most important physiological processes inside the body [6]. Apoptosis is responsible for the regulation of the immune response, tissue development and homeostasis. This type of cell death is controlled by triggering several signaling molecules, protein–protein interactions, binding receptors and genes encoded in their pathways to stimulate specific signaling proteins and to produce the required energy for cell work [7]. One such protein involved in the apoptosis pathway is cytochrome C. Cytochrome C is released from the mitochondria after apoptotic stimulation and produces a downstream effect, which ultimately results in programmed cell death [8]. Hence, introduction of cytochrome C into tumour cells is likely to result in apoptosis. However, due to the relative size (12.7 kDa) of the protein, intracellular delivery is not possible without the use of excipients [9]. Over the past decade, multiple nanotechnologies have been developed to act as transfection agents, carrying proteins across the cell membrane or permeating across tissue [9,10]. A relatively new type of nanoparticle that has been reported with potential in this area is hybrid iron oxide-gold nanoparticles (HNPs) [11,12]. HNPs have been reported to be efficient as drug carriers in pancreatic cancer therapy, where they guide the drug of choice into the cell via endocytosis—thus resulting in larger quantities of internalised drugs compared with the unformulated molecules [13,14]. Previously, we have reported that cytochrome C can become irreversibly bound onto the surface of these HNPs via dative covalent linkage between the gold surface of the HNP and a thiol (-SH) residue in the cytochrome C [12]. We showed that increased intracellular concentration of cytochrome C was evident after conjugation onto hybrid iron oxide-gold nanoparticles (HNPs) [12]. These increased levels compared with the native unbound protein were observed at varied incubation concentration (0.2 mg·mL^−1^ and 0.5 mg·mL^−1^) in a concentration dependant manner; however, measurements after 1 h and 4 h exposure showed that time dependent uptake did not occur [12]. Additionally, the data showed that increased intracellular levels of cytochrome C in combination with doxorubicin resulted in an increased therapeutic effect. The exact mechanism of how this is achieved is unknown due to the permanent binding of cytochrome C onto the HNP surface (HNP-c). Irrespective of the HNP-c being a stable system, it was postulated that the tightly bound cytochrome C was still able to influence the apoptosis pathway [12].

As knowledge of cancer physiology and treatment has advanced, it has become evident within the past decade that combined therapies have many advantages over single therapy [15]. Although more costly, the clinical outcome is more impactful [16]. This is due to the ability of combined therapies to cause a ‘double blow’ or synergy to tumour cells by interrupting more than one cellular function, hence causing a sense of cellular vulnerability and greater potential for maximum efficacy. Hence, we postulate that combining the delivery of cytochrome C into the cancer cells as well as dosage of chemotherapy will result in a highly cytotoxic system whereby greater levels of apoptosis and cellular mortality are observed, making this a potential treatment for this devastating disease. Hence, in this work, we investigate the combined effect of pro-apoptotic cytochrome C (conjugated onto HNPs) with a panel of chemotherapy agents (doxorubicin, paclitaxel oxaliplatin, vinblastine sulfate salt and vincristine sulfate salt) on various liver cancer cell lines for the treatment of HCC. 

## 2. Materials and Methods 

Doxorubicin, paclitaxel, oxaliplatin, vinblastine sulfate salt and vincristine sulphate salt were purchased from LC Laboratories (Woburn, MA, USA); cytochrome C and other materials were purchased from Sigma-Aldrich (Gillingham, UK). Hepatocellular carcinoma cell line (HepG-2) was provided from American Type Culture Collection (ATCC) (ATCC^®^ HB8065™), SK-hep-1 was from ATCC (ATCC^®^ HTB-52™) and Huh-7D cell line was from ECACC (European Collection of Authenticated Cell Cultures).

### 2.1. Synthesis and Decoration of Hybrid Nanoparticles

Hybrid nanoparticles (HNPs) were synthesised and characterised as previously described [17,18]. Briefly, iron oxide nanoparticles were fabricated through a precipitation reaction and stabilised with poly(ethylenimine) (*M*_W_ 200,000). Iterative gold coating was achieved by reduction of tetrachloroauric acid onto gold seeding on the HNP surface. Cytochrome C (25 mg) was conjugated irreversibly onto the nanoparticle surface (5 mL, 5 mg·mL^−1^) via dative covalent binding of the thiol residue in the protein structure with the gold surface of the HNP as reported previously [12].

### 2.2. Subculture of Cell Lines

The cell lines were grown as a monolayer in ATCC-formulated Eagle’s Minimum Essential Medium for (SK-hep-1); and DMEM culture media for HepG-2 and Huh-7D, and placed in a humidified incubator with 5% CO_2_ at 37 °C. All growth medium was supplemented with 10% fetal bovine serum (FBS) and 1% penicillin streptomycin.

### 2.3. Cellular Uptake of Cytochrome C

Liver cell lines were seeded in 6-well plates (50,000 cells/well) and incubated for 24 h at 37 °C with 5% CO_2_. The media was removed and replaced with 100 µL (50 µg·mL^−1^ or 100 µg·mL^−1^) of cytochrome C or HNP-c and incubated for 1 h or 4 h. The media was removed and the treated cells washed with PBS and trypsinised. The cells were suspended in 1 mL fresh media and were counted using an automated cell counter (Invitrogen Countess, Carlsbad, CA, USA). Afterwards, 100,000 cells were placed into an Eppendorf tube and centrifuged for 5 min at (750 rpm). The resultant supernatant was removed and the precipitated pellets were resuspended with 1 mL of sterilised water. The concentration of protein alone and in combination with HNP was measured at 410 nm and analysed per cell by using UV/Vis spectroscopy (UV-VIS with an ISR-2600 Shimadzu, Duisburg, Germany). The amount of cytochrome C per cell was estimated by dividing the concentration determined by the cell number (100,000).

### 2.4. Cell Viability Evaluation

#### 2.4.1. MTT Assay

Cells were seeded into 96-well plates (15,000 cells/well) and incubated overnight. Serial dilutions of doxorubicin (0.25 μM–5 μM); oxaliplatin (8 μM–128 μM); paclitaxel (1 nM–100 nM); vincristine and vinblastine (1 nM–100 nM) alone and in combination with HNP-c (IC_10_ concentration previously determined in HepG2 [12] and fixed at 0.012 mg·mL^−1^) were added to the 96 wells of HepG2, SK-hep-1 and Huh-7D cell lines and incubated for (24 h, 48 h and 72 h). After this time, the media was removed, cells were washed with fresh media and 100 μL of 3-(4,5-dimethyl-2-thiazolyl)-2,5-diphenyl-2*H*-tetrazolium bromide) (MTT) was added to the cells. The plates were incubated for 4 h. The MTT solution was removed and replaced by 100 μL of DMSO. PBS and Triton X (80 µL each) were used as the negative and positive controls, respectively. The absorbance of each well was measured at 570 nm using a Tecan Infinite Pro microplate reader (Tecan, Männedorf, Switzerland). Percentage cell viability was calculated relative to positive and negative controls. Experiments were carried out in triplicate.

#### 2.4.2. Trypan Blue Exclusion

Cells were seeded into 6-well plates (50,000 cells/well) and incubated overnight. Serial dilutions of doxorubicin (0.25 μM–5 μM); oxaliplatin (8 μM–128 μM); paclitaxel (1 nM–100 nM); vincristine and vinblastine (1 nM–100 nM) alone and in combination with HNP-c (IC_10_ concentration previously determined in HepG2 [12] and fixed at 0.012 mg·mL^−1^) were added to the 6-wells of HepG2, SK-hep-1 and Huh-7D cell lines and incubated for (24 h, 48 h and 72 h). After this time, the cells were washed 5 times with PBS before being trypsinated and resuspended in 1 mL fresh media. The cell suspension (50 µL) was mixed with 50 µL of trypan blue and live cells counted using an automated Countess™ (Invitrogen, CA, USA). PBS (80 µL each) was used as the controls respectively. The percentage of viability was calculated in relation to control cells.

### 2.5. Apoptosis Detection

#### 2.5.1. Caspase-3 Assay

Cells were seeded into 6-well plates (50,000 cells/well) and incubated overnight. Cells were treated with 5 times their IC_50_ concentration (as per manufacturer protocol, Abcam, Cambridge, UK) of doxorubicin, paclitaxel, oxaliplatin, vincristine and vinblastine alone and in combination with HNP-c at the IC_10_: 10% inhibition concentration. Plates were incubated for 24 h. The media was removed and the cells were trypsinised and resuspended in fresh media. The cell suspensions were centrifuged for 10 min at 250 g. The supernatant was carefully removed and 25 μL of cold lysis buffer was added to the cell pellet. The assay buffer and dithiothreitol (DDT) stoke solution was added to the cells in accordance with the manufacturer instructions (Abcam, Cambridge, UK). Caspase-3 colorimetric substrate (Ac-DEVD-pNA) (5 μL) was added to the cells. The solution was then transferred into a 96-well plate and incubated at 37 °C for 2 h. After incubation, the absorbance of the wells was read at 405 nm using a Tecan Infinite Pro microplate reader (Tecan, Männedorf, Switzerland). The extent of apoptosis was measured in relation to positive and negative controls (without substrate as positive and without cells as negative as per assay protocol). Experiments were carried out in triplicate.

#### 2.5.2. Western Blotting for Detection of Poly (ADP-ribose) polymerase (PARP)

Cells were seeded into 6-well plates (1 × 10^6^ cells/well) and incubated for 48 h. Cells were treated with 5 times their IC_50_ concentration (as per PARP detection protocol) of doxorubicin, paclitaxel, oxaliplatin, vincristine and vinblastine alone and in combination with HNP-c at the IC_10_. After this time, the media was removed, the cells were trypsinised and resuspended in fresh media. The cell suspension was centrifuged for 3 min at 750 rpm, at 4 °C. The cell pellets were washed with chilled PBS and centrifuged again and the resultant cell pellets were cooled to −80 °C. The chilled cell pellets were collected and lysed by adding 100 µL of RIPA (RIPA buffer is used to lyse cells and tissues. 1× RIPA Buffer: 20 mM Tris-HCl (pH 7.5), 150 mM NaCl, 1 mM Na_2_EDTA, 1 mM EGTA, 1% NP-40, 1% sodium deoxycholate, 2.5 mM sodium pyrophosphate, 1 mM β-glycerophosphate, 1 mM Na_3_VO_4_ and 1 µg·mL^−1^ leupeptin) buffer and centrifugation for 10 min at 14,000 rpm, 4 °C, the resultant supernatant was collected and stored at −80 °C. 

The bicinchoninic assay (BCA) was used as quantitative method to detect the protein concentration for each sample by mixing 10 µL of cell lysate with 100 µL of BCA reagent and incubating for 30 min at 37 °C. The absorbance of the samples was read at 570 nm using a Tecan Infinite Pro microplate reader (Tecan, Männedorf, Switzerland) and compared with a BSA standard.

Each sample was mixed with 5 µL of prepared buffer of (β-mercaptoethanol and SDS-Nupage, 1:20) and boiled at 80 °C before gentle centrifugation. The samples were transferred into the wells of a 4–20% Bis Tris gel (Fisher Scientific, Loughborogh, UK) and electrophoresis was carried out. Subsequently, the gel proteins were transferred electrically to the PVDF membrane soaked in transfer buffer solution. The blocking process was performed to the PVDF protein membrane by using milk solution (comprised of 5 g of semi-skimmed dry milk and 100 mL Tris buffer saline tween) (TBST 1×) for 1 h at room temperature. The primary detected antibody (PARP) in ratio of 1:2000 was prepared and soaked with the PVDF membrane overnight. The membranes were washed and incubated with TBST 1×, followed by another incubation period with a secondary antibody that was prepared by mixing anti-rabbit AB with blocking milk solution in a dilution ratio of 1:2500, respectively. An ECL imaging detecting system was used to detect the reactive protein bands. Experiments were carried out in triplicate.

#### 2.5.3. Terminal Deoxynucleotidyl Transferase (dUTP) Nick End Labelling (TUNEL) Assay

A TUNEL colorimetric method was used to detect the DNA damage marker for apoptosis detection in 96-well plate kits that were purchased from (R&D System, Minneapolis, MN, USA). Cell lines were cultured in 96-well plates with concentration (50,000 cells/well) and then treated with doxorubicin, paclitaxel, oxaliplatin, vinblastine or vincristine alone and in combination with HNP-c (IC_10_). The plates were incubated for 24 h. The media was removed from each well and the cells were fixed with 3.7% buffered formaldehyde solution for 7 min, followed by washing with PBS (2 times) and centrifugation between each washing step. The post-fix step was performed by filling the wells with 100% methanol for 20 min and washing with PBS before labelling. After which, 100 μL/well of 5% phosphoric acid was added to stop the reaction and the absorbance of the wells was measured at 450 nm using a Tecan Infinite Pro microplate reader (Tecan, Männedorf, Switzerland). Positive and negative controls were generated according to the protocol to indicate the level of DNA breakage generated for the labelling reaction as well as the unlabelled control to show the background of labelling processes. Experiments were carried out in triplicate.

### 2.6. Imaging Studies

#### 2.6.1. Fluorescent Microscope

Cell lines were seeded onto glass cover slip in 6-well plates (50,000 cells/well) and incubated for 24 h. Doxorubicin (5 μM); oxaliplatin (128 μM); paclitaxel (100 nM); vincristine and vinblastine (100 nM) alone and in combination with HNP-c (0.012 mg·mL^−1^ or 1 µM) were added to the cell lines and incubated for 48 h. The growth media was removed from each well and the cells were washed three times with 1 mL of 1× annexin binding buffer. Annexin V fluorescein conjugate (5 µL), 5 µL of Hoechst nuclei stain and 5 µL of propidium iodide per 100 µL of 1× annexin binding buffer was added to each well and incubated at room temperature for 15 min and protected from light. The washing process was repeated three times with 1 mL of 1× annexin binding buffer. The cells were fixed with 2% formaldehyde for 10 min and washed three times with PBS. The glass slips were fixed on the glass slides and visualised by fluorescence microscopy (Invitrogen, EVOS^TM^ FL, Carlsbad, CA, USA).

#### 2.6.2. Nanoparticle Internalisation Imaging

Cross section imaging of the nanoparticles inside the cells were imaged using transmission electron microscopy (TEM, JEOL, Tokyo, Japan). Cells were seeded onto Aclar slides within 6-well plates (50,000 cells/well) and incubated with 25 µg·mL^−1^ of HNP-c for 24 h. The cells were washed with PBS followed by fixation with 2.5% gluteraldehyde in a sodium cacodylate:calcium chloride buffer for 2 h. The cells were washed with the buffer for 5 min and this process was repeated a further two times. Post fixation 0.1% osmium tetroxide in 0.1 M sodium cacodylate buffer: 2 mM Ca chloride was incubated with the cells for 1 h. Dehydration with serial dilutions of ethanol was carried out before infiltration of samples within Spurr resin. The ultra-sectioning process of resin was achieved by a diamond knife and the ultrathin sections were placed onto copper grids for staining with heavy metals. The cells were imaged on a JEOL, JEM-1230 microscope (JEOL, Tokyo, Japan).

## 3. Results and Discussion

The development of novel drug treatment strategies is crucial in order to increase the outlook for patients diagnosed with liver cancer. One of these strategies is to deliver pro-apoptotic cytochrome C into tumour cells to stimulate apoptosis. This could offer a significant increase in the efficacy of chemotherapeutic drugs by working in synergy with a mechanism of drug action [19,20]. The delivery of the cytochrome C mediated by hybrid nanoparticles offers many advantages due to the multifunctional nature of the particles. Not only can the gold surface be easily decorated with cytochrome C and targeting ligands, but also the magnetic properties of the iron oxide core may be directed to the specific site required in the body by applying the external source of the magnetic field. By this application, we can get a high aggregation of the bio-activated magnetic nanoparticles inside tumour cells [21,22].

### 3.1. Cellular Internalisation

The HNPs were successfully synthesised and surface conjugated with cytochrome C as previously reported. The ratio of Fe:Au:cytochrome C was 1:0.2:0.45. All subsequent concentrations reported in this paper when referring to HNP-c will be in relation to the cytochrome C concentration.

**Figure 1:**
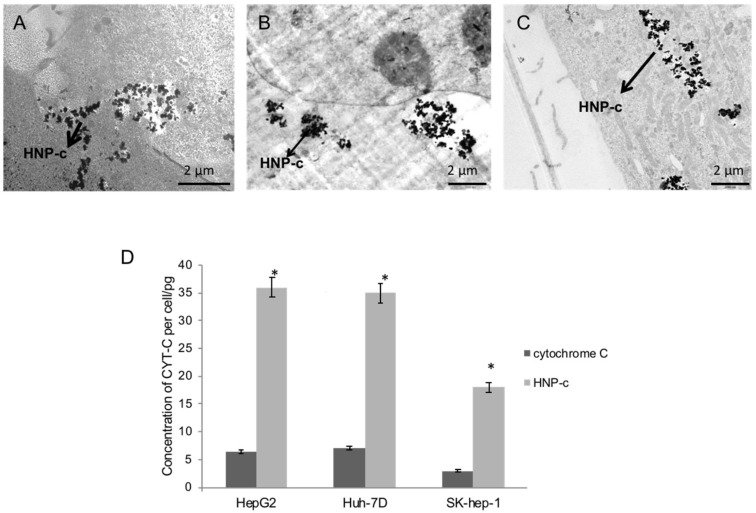
Cellular internalisation of HNP-c (hybrid nanoparticles with cytochrome C conjugated onto the surface) imaged using TEM (transmission electron microscopy) after exposure to (**A**) HepG2, (**B**) Huh-7D and (**C**) Sk-hep-1 cells with (**D**) quantification using ICP-OES (inductively coupled plasma − optical emission spectroscopy, *n* = 3, ±SD). * denotes significant increase compared to cytochrome C alone (*p* < 0.05).

The TEM micrographs in Figure 1 show the uptake of the HNP-c into (A) hepatocellular carcinoma (HepG2), (B) epithelial hepatoma (Huh-7D) and (C) endothelial hepatocellular carcinoma (SK-hep-1) cell lines. Here, it is evident that the nanoparticle formulation is internalised into the cellular cytoskeleton via endocytosis, before being released from the endosomes into the cytoplasm. The cell lines were incubated with 50 µg·mL^−1^ and 100 µg·mL^−1^ of HNP-c and the internalised amount of cytochrome C present was calculated per cell (Figure 1D). Consistent with our previous findings (using only HepG2 cells [12]), the protein internalisation level was higher after conjugation onto the HNPs compared with free protein with a 5.5-fold, 5-fold and 6-fold improvement in internalisation levels at 100 µg·mL^−1^ incubation for 4 h in the HepG2, Huh-7D and SK-hep-1 cells, respectively. Interestingly, the internalisation both with the free protein and the bound HNP-c in SK-hep-1 cells showed considerably lower levels compared with the other two cell lines, perhaps this is due to the smaller cellular volume hindering further uptake; nevertheless, the -fold improvement after conjugation onto the HNPs was consistent with the other cell lines. The uptake experiments showed a concentration dependent pattern of uptake (*p* < 0.05). However, no long-term time dependency was observed in terms of intracellular concentration detected, whereby, after 1 h, the cellular uptake appeared to plateau and no further increase in intracellular concentration was observed after 4 h in agreement with previous findings [12].

### 3.2. Cytotoxicity

**Figure 2:**
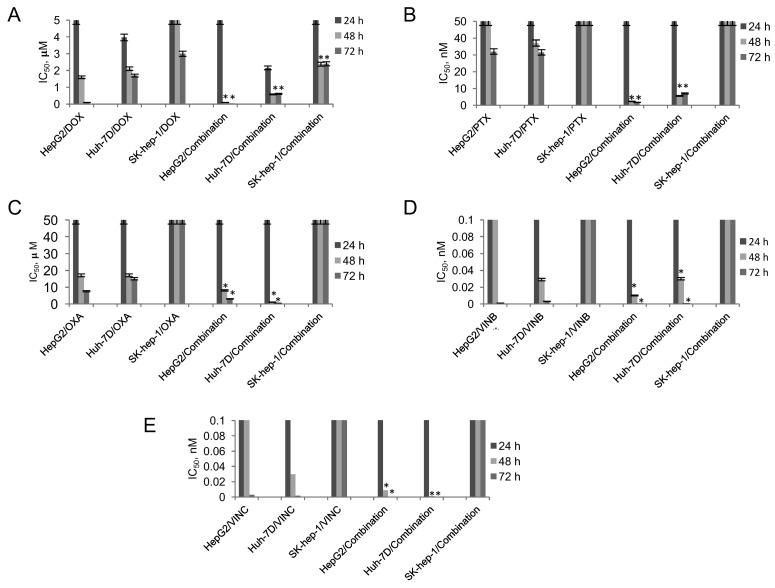
Cell viability measured using the MTT assay of (**A**) doxorubicin, (**B**) paclitaxel, (**C**) oxaliplatin, (**D**) vinblastine and (**E**) vincristine as a single therapy and in combination with HNP-c after 48 h incubation in HepG2, Huh-7D and SK-hep-1 cell lines (*n* = 3, ±SD). * denotes significant decrease in IC_50_ compared to single drug treatment (*p* < 0.05).

The cytotoxic effect of the combined treatment of the liver cancer cell lines in combination with a range of chemotherapeutic agents (doxorubicin, paclitaxel, oxaliplatin, vinblastine and vincristine) was determined compared to the single dosage over a 72 h period. Figure 2A shows the results from the MTT assay on the cell lines treated with doxorubicin. Here, no IC_50_ was observed after 24 h incubation in all cell lines, both after exposure to drugs alone as well as in combination with HNP-c. At increased exposure times, IC_50_ values of 1.6 µM and 0.1 µM were observed after 48 h and 72 h with doxorubicin treatment. After combination treatment with the HNP-c, a 15-fold and 10-fold reduction in IC_50_ was observed. Consistent with the MTT results in HepG2 cells, the Huh-7D cells showed a similar trend, whereby, an increase in cytotoxicity (decrease in IC_50_) was consistently observed in combination treatment after all time points (24 h: 0.75-fold, 48 h: 2.6-fold and 72 h: 1.7-fold). The SK-hep-1 cell lines were much more resistant to drug treatment; here, no IC_50_ was observed until 72 h exposure (3 µM). In combination treatment, a comparable IC_50_ was observed after only 48 h, which did not reduce further upon longer incubation. 

**Figure 3:**
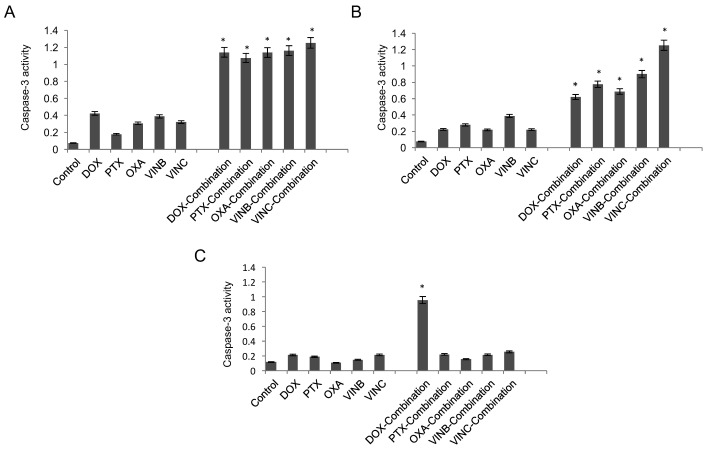
Apoptosis detection via Caspase-3 measurement in (**A**) HepG2, (**B**) Huh-7D and (**C**) SK-hep-1 cells incubated with chemotherapies both with and without HNP-c (*n* = 3, ±SD). Control refers to cells with no treatment. * denotes significance compared to single drug treatment (*p* < 0.05).

Figure 2B shows the MTT data for the cell lines in drug treatment and combination treatment with paclitaxel. The data shows that, for paclitaxel in HepG2 cell lines, no IC_50_ was observed until 72 h treatment (32 nM), in those cells treated in combination with HNP-c, an IC_50_ was evident at 48 h, which was much lower at 2.3 nM. The Huh-7D cells showed reduction in viability after only 24 h drug treatment, with IC_50_ values reduced by 5.6-fold after combination therapy. A similar trend was observed across all incubation times where a greater cytotoxic effect was observed after combination therapy. In the SK-hep-1 cell lines, no IC_50_ values were observed both in single drug treatment and in combination therapy with HNP-c at all the time points tested. Cell lines treated with oxaliplatin (Figure 3C) showed IC_50_ values after 48 h and 72 h in both HepG2 and Huh-7D cell lines. However, combination treatment with this drug and HNP-c did not appear to result in a greater cytotoxic effect compared to drugs alone. Drug treatment with vinblastine (Figure 2D) did not show any great cytotoxic effect until 72 h exposure in the HepG2 cells, whereas, in Huh-7D cell lines, an IC_50_ was observed after 48 h. Consistently, after 72 h, a 9-fold increase in cytotoxicity was observed after 72 h in both cells. In the SK-hep-1 cell lines, no effect was observed in both the drug alone and combination therapy across all time points. A similar trend was observed in cells treated with vincristine (Figure 2E) and vinblastine (Figure 2D) with a 29-fold and 19-fold decrease in IC_50_ after 72 h in HepG2 and Huh-7D cells that underwent combination therapy. In addition, no major reduction in cell viability was observed in SK-hep-1 cells.

The cytotoxicity was also measured using trypan blue counting, in the HepG2 (Table S1), Huh-7D (Table S2) and SK-hep-1 (Table S3) cells. This data in general showed similar trends and was in agreement with the MTT assay data.

In general, the SK-hep-1 cell line exhibited a much greater degree of drug resistance compared to the other cell lines tested. We believe this may be due to the fact that they do not possess any mRNA of hepatic-specific proteins (albumin and fibrinogen) or due to a difference in proliferation rate or cellular uptake mechanism of drugs/particles compared with the other cell lines [23]. Hence, in these highly resistant cells, we did not observe any IC_50_ when incubated with all the drug molecules except doxorubicin. Doxorubicin has been classified as an intracellular and extracellular affecting chemotherapeutic drug (non-specific cell cycle phase killing drug) [24]. Hence, this may render it more effective in drug resistant cells rendering the effect we observed.

### 3.3. Apoptosis Detection

#### 3.3.1. Caspase-3 Assay

Cascade signalling pathways are involved in programmed cell death by activation of specialised caspase families. When inactive precursors of these enzymes are activated by extrinsic and/or intrinsic apoptosis pathways, a series of cleaving and stimulating steps are directed to the effector and executioner groups of the caspases to initiate apoptosis [25]. Caspase-3 is one of the executioner caspase members and its expression can be measured in order to give an indication of apoptosis levels in the cells. The capase-3 levels for HNP-c treated cells were tested on all cell lines at the concentrations tested for combination therapy and did not result in any increase in activity compared with the control (cells with no treatment). Figure 3A shows the capase-3 data in HepG2 cells. Here, caspase levels were significantly increased in those cells treated in combination therapy with all drug molecules. Combination treatment resulted in a 2-fold, 5.2-fold, 2.6-fold, 2.8-fold and a 3-fold increase in enzyme levels compared to the drug alone (doxorubicin, paclitaxel, oxaliplatin, vinblastine and vincristine respectively). Consistent with this data, the caspase-3 enzyme levels in the Huh-7D were increased after combination therapy compared to the single drug treatment (Figure 3B) with a similar level of increase as observed in the HepG2 cells. For the SK-hep-1 cell line, no significant results were obtained in caspase-3 assay with cells treated with drugs alone or in combination therapy (Figure 3C), with one exception. In those cells treated with doxorubicin in combination with HNP-c, a 3.5-fold increase in caspase-3 levels was observed compared with the drug alone. These findings were in agreement with the cytotoxicity data, whereby doxorubicin was the only drug that exhibited an IC_50_ value both as a single agent and in combination therapy (Figure 2A). However, differing from the cytotoxicity data, significant levels of caspase-3 were only evident in combination therapy, and not in those cells undergoing drug treatment alone. The reason for this is not yet known.

#### 3.3.2. Western Blotting for Detection of PARP

**Figure 4:**
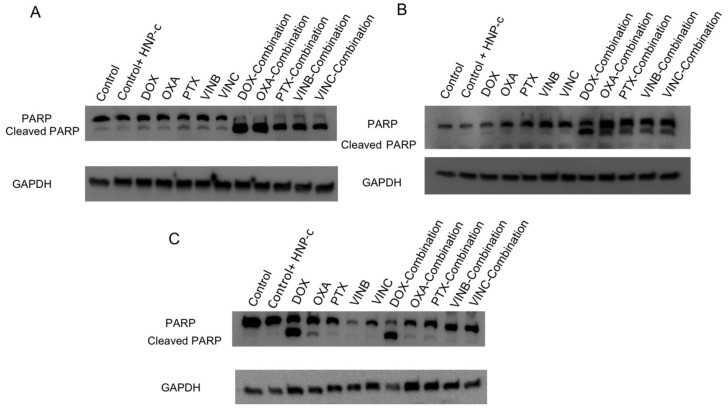
Western blot detection of poly (ADP-ribose) polymerase (PARP) cleavage in (**A**) HepG2, (**B**) Huh-7D and (**C**) SK-hep-1 cell lines incubated with chemotherapy both with and without HNP-c. Control refers to cells with no treatment.

Apoptosis is mediated by the caspase series family by the cleavage of many key proteins used in the proper cell growth pathway [26]. One of the cellular caspase substrates is poly(ADP-ribose) polymerase (PARP). Cleavage fragments of this protein are considered as the main marker of apoptosis. Hence, cleavage PARP bands in Western blots are recognised as the main biomarkers of programmed cell death by apoptosis, which follows a unique pattern of protease enzymes activity. Figure 4 shows the results of the Western blots carried out using (A) HepG2, (B) Huh-7D and (C) S-hep-1 cells. In HepG2 cells, no PARP cleavage bands were detected in those cells incubated with either HNP-c or anticancer agents (Figure 4A). However, PARP cleavage bands were evident in cells treated in combination therapy, with stronger bands evident in those cells treated with both doxorubicin or oxaliplatin in combination with HNP-c. In Figure 4B, faint PARP cleavage bands can be observed across all single drug treatments. Those cells treated in combination therapy showed dark PARP cleavage banding consistently across all anticancer agents, indicating increased levels of apoptosis. In Figure 4C, the SK-hep-1 cells showed the appearance of dark PARP cleavage bands in only those samples treated with doxorubicin alone or doxorubicin combination therapy. This data is in agreement with the cytotoxicity data where doxorubicin was the only drug that exhibited any IC_50_ value in those cells. A faint PARP cleavage band was observed in those cells treated with oxaliplatin; however, this becomes more faint in those cells treated with combination therapy.

#### 3.3.3. Terminal Deoxynucleotidyl Transferase (dUTP) Nick end Labelling (TUNEL) Assay

DNA fragmentation is one of most important features of apoptosis. One of these methods is the labelling of the nick end of damaged DNA with TDT-mediated dUTP-biotin [27]. The TUNEL assay is used for the quantitative detection of apoptotic cells at the late stage by dUTP labelling of the free 3-hydroxy termini of DNA breaks, which is catalysed by the presence of the TDT enzyme. 

**Figure 5:**
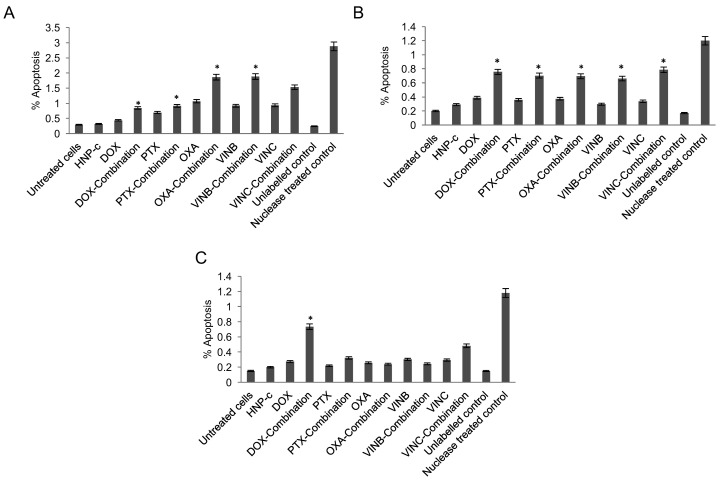
Apoptosis detection via TUNEL assay measurement in (**A**) HepG2, (**B**) Huh-7D and (**C**) SK-hep-1 cells incubated with chemotherapies both with and without HNP-c (*n* = 3, ±SD). * denotes significance compared to single drug treatment (*p* < 0.05).

Figure 5A shows the percentage of apoptotic labelled HepG2 cells. For all the combination therapies tested, a significant increase in the percentage of apoptosis was observed compared with treatment of drug alone. This result was in agreement with the caspase-3 (Figure 3A) and Western blot data (Figure 4A). Consistently, Figure 5B follows a similar trend, whereby combination therapy does indeed result in an increase of apoptosis levels. Figure 5C shows the apoptosis levels in the SK-hep-1 cell line after treatment. In agreement with the results from the caspase-3 assay (Figure 3C), only those cells treated with doxorubicin in combination with HNP-c resulted in a significant increase (2.7-fold) in the level of apoptosis compared with untreated cells. 

### 3.4. Fluorescent Microscopy

**Figure 6:**
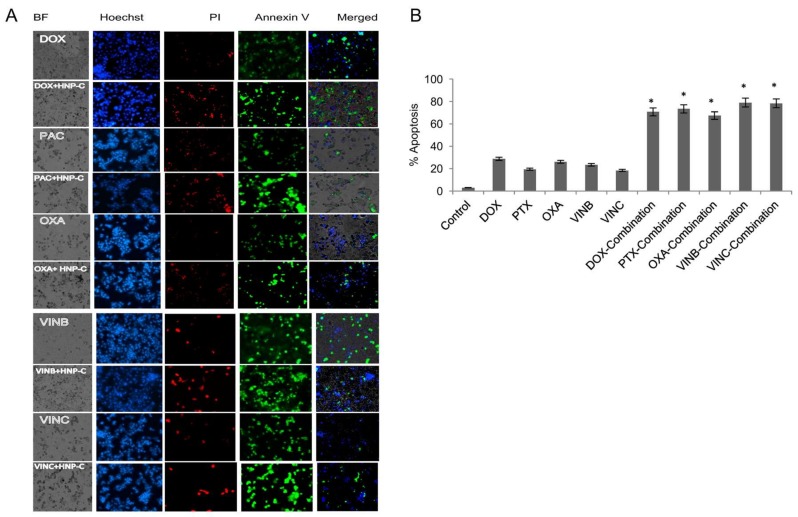
Apoptosis detection cells with Annexin V staining probe. HepG2 cells treated with single drugs and in combination with HNP-c demonstrated as (**A**) images and (**B**) quantitatively (*n* = 3, ±SD). Control refers to cells with no treatment. * denotes significance compared to single drug treatment (*p* < 0.05).

Apoptosis is characterised by several changes to the cellular components such as a condensed nucleus, DNA fragmentation and morphological changes of cell surfaces. Visualising the different stages of apoptosis is possible using different methods. In early stage apoptosis, the phosphatidylserine (PS) is translocated from the inner to the outer face of the cell membrane, which can be visualised by binding fluorescein isothiocyanate labelled annexin V (annexin V-FITC) to the PS. The apoptotic cells are then visualised as a green staining on the outer membrane. For late stage apoptosis, propidium iodide (PI) was used in a fluorescence binding assay that had the ability to penetrate only compromised cell membranes and was visualised as a red colour under the microscope. The condensed nucleus can be further detected as blue staining nuclei with Hoechst fluorescence dye. Figure 6A shows the fluorescent microscopy imaging of HepG2 cells after both drug and combination treatment with HNP-c. In HepG2 cells, all the cells treated with a single drug dose showed high levels of annexin V.

Those cells treated with combination therapy showed high levels of both PI and annexin V. The cells treated with doxorubicin combined with HNP-c showed a 2.5-fold increase in the percentage of early apoptotic cells compared with drugs alone (Figure 6B). The level of annexin V also increased for paclitaxel (3.8-fold), oxaliplatin (2.6-fold), vinblastine (3.4-fold) and vincristine (4.3-fold) after combination treatment compared to drugs alone. Similar results were found in the Huh-7D cells (Figure S1). Consistent with the apoptosis studies, in SK-hep-1 cells (Figure S2), only those cells incubated with doxorubicin in combination showed a significant increase in annexin V levels, although the presence of annexin V was evident in all the cell treatment groups at low levels.

Overall, our findings are considered as a smart alternative to current hepatocellular carcinoma treatments. The apoptotic effects of the HNP-c, solo drug and combined therapies have been investigated at the different apoptotic stages. The relative caspase-3 activity was measured as a result of cytochrome C binding to Apaf-1 and pro-caspase 9 to form apoptosome and consequently start the apoptotic signal of caspase cascade pathway [28]. As described, the combination of chemotherapeutics and HNP-c resulted in a 3–4 fold increase in activation levels of caspase-3 relative to single drug treatments.

Apoptosis induction was also confirmed by protein binding visualization. The most detectable cleavage nuclear protein poly (ADP-ribose) polymerase-1 (PARP) was used as a main apoptotic marker in bio-therapeutic treatments. Studies have reported the covalent binding of cytochrome C onto an antennapedia (Antp) peptide in Hela cell lines. Significant PARP cleavage was observed in those cells exposed to cytochrome C binding to Antp due to the cleavage of the 116 KDa PARP-1 precursor into 89 KDa PARP-1 fragments. This phenomenon was not observed in those cells treated with either cytochrome C or Antp alone where no PARP cleavage was reported [20,29,30]. In our study, we observed obvious cleavage bands after cell treatment with the combination therapies compared to faint banding detected with the single drug treatments. This was in agreement with the caspase-3 assay. Further studies were carried out to detect the apoptotic effects of the combination therapies using DNA 3-OH labeling kits (TUNEL assay). As shown, the quantification methods of TUNEL assay showed a significant increase in the absorbance levels of the combination therapy compared with the single drug treated cells. 

Additionally, fluorescent binding (Annexin) probes were used to detect the early stages of apoptosis by labeling the external cell membrane folding protein in apoptotic cells phophtidylserine (PS), which could be differentiated from late apoptosis and necrotic cells that permitted the fluorescent propidium iodide (PI) probes to penetrate the cell membrane. Apoptosis induction in A549 cells has previously been reported when exposed to cytochrome C loaded formulations compared to the free protein as imaged under confocal microscope. Here, green fluorescent Annexin-V signals were observed due to early stage apoptosis and red fluorescent signals were observed due to late apoptotic cells [31]. Similarly in our studies, we were able to visualise the presence of early apoptosis after combination treatment, demonstrating the more rapid and effective treatment of the co-therapy approach.

Another fluorescent study is also reported with HepG2 incubated with doxorubicin alone and after binding onto hollow mesoporous silica nanoparticles (HMSNs) grafted with cytochrome C [32]. The authors showed that the HMSN nanoparticles grafted with doxorubicin and cytochrome C exhibited significantly increased apoptosis levels compared with either the nanoparticle alone or with doxorubicin treatment [32]. These findings are in agreement with our studies, whereby the HNP-c and doxorubicin combination treatment was shown to potentiate the apoptotic activity in a dose dependent manner.

## 4. Conclusions

This work focused on the use of a range of anticancer agents in combined therapy with HNP-c for liver cancer treatment. The data showed consistently across the HepG2 cells and Huh-7D cells that combined treatment resulted in a synergistic effect whereby there was greater apoptosis generation and an increase in cytotoxicity with combination therapy compared to the drug alone. The cytotoxicity assays showed that the anti-microtubule drugs (paclitaxel, vinblastine and vincristine) were generally more cytotoxic, with lower IC_50_ values, than the DNA damaging drugs (doxorubicin and oxaliplatin). The SK-hep-1 cells were much more resistant to treatment and, in fact, although not the most potent drug, doxorubicin consistently outperformed the other drugs both alone and in combination therapy, showing an enhanced cytotoxic effect. The data presented confirms the hypothesis that, by introducing the pro-apoptotic protein cytochrome C into the cells and co-administering with anticancer agents, a synergistic effect will occur, resulting in increased cellular death. This exciting finding indicates that administration of cytochrome C in parallel with clinically used chemotherapies may result in more favourable patient outcomes at a relatively low cost. The work from this study will be used to inform an in vivo trial on xenograft models in order to determine whether this in vitro phenomenon may translate in vivo in order to prove clinically relevant. Additionally, it would be advantageous moving forward to formulate the anticancer agents alongside the chemotherapy in order to perform one single co-administration dose. Unfortunately, most anticancer agents are water insoluble and therefore could not be conjugated onto the HNP with good levels of stability. Hence, in order to push this formulation technology forward, it is necessary to design a larger supramolecular structure capable of encapsulation of the hydrophobic agents. 

## Supplementary Materials

The following are available online at http://www.mdpi.com/1999-4923/10/2/48/s1, Figure S1: Apoptosis detection cells with Annexin V staining probe. Huh-7D cells treated with single drugs and in combination with HNP-c demonstrated as A) images and B) quantitatively (*n* = 3, ±SD), Figure S2: Apoptosis detection cells with Annexin V staining probe. SK-hep-1 cells treated with single drugs and in combination with HNP-c demonstrated as A) images and B) quantitatively (*n* = 3, ±SD), Table S1: IC_50_ values of drugs and drug combinations tested in HepG2 cell lines over 72 h, Table S2: IC_50_ values of drugs and drug combinations tested in Huh-7D cell lines over 72 h, Table S3: IC_50_ values of drugs and drug combinations tested in SK-hep-1 cell lines over 72 h.
