# Supplementary material for: Combined Effect of Anticancer Agents and Cytochrome C Decorated Hybrid Nanoparticles for Liver Cancer Therapy
Source: Pharmaceutics. 2018 Apr 12;10(2):48. doi: 10.3390/pharmaceutics10020048 (PMC6027273; doi:10.3390/pharmaceutics10020048)
Supplement: Supplementary file 1 [file pharmaceutics-10-00048-s001.pdf]

Supplementary Materials: Combined Effect of Anticancer Agents and Cytochrome C Decorated Hybrid Nanoparticles for Liver Cancer Therapy

Wejdan Al-Shakarchi, Ali Alsuraifi, Mohammed Abed, Marwan Abdullah, Alan Richardson, Anthony Curtis and Clare Hoskins

Table S1. IC_{50} values of drugs and drug combinations tested in HepG2 cell lines over 72 h.

	**IC_{50} (drug) MTT**	**IC_{50} (drug-combination) MTT**	**IC_{50} (drug) Trypan blue**	**IC_{50} (drug-combination) Trypan blue**
		**DOXRUBICIN**		
**24 h**	NS	NS	NS	NS
**Increase in cytotoxicity**	-	-		
**48 h**	1.6 ± 7.5 μM	0.1 ± 2.34 μM	1.46 ± 1.14μM	0.09 ± 1.87 μM
**Increase in cytotoxicity**	-	15-fold		15.2-fold
**72 h**	0.1 ± 4.69 μM	0.009 ± 0.38 μM	0.27 ± 3.54 μM	0.002 ± 2.82 μM
**Increase in cytotoxicity**		10.1-fold		12.5-fold
		**PACLITAXEL**		
**24 h**	NS	NS	NS	NS
**48 h**	NS	2.3 ± 2.09 nM	NS	1.60 ± 3.53 nM
**Increase in cytotoxicity**	-	Significant IC_{50}		Significant IC_{50}
**72 h**	32 ± 4.84 nM	1.7 ± 1.97 nM	35.72 ± 1.41 nM	0.18 ± 4.77 nM
**Increase in cytotoxicity**	-	17.8-fold		193-fold
		**OXALIPLATIN**		
**24 h**	NS	NS	NS	NS
**Increase in cytotoxicity**	-	-		
**48 h**	17 ± 1.56 μM	8 ± 4.63 μM	25.21 ± 2.82 μM	6.510 ± 1.41 μM
**Increase in cytotoxicity**	-	1.1-fold	-	2.8-fold
**72 h**	7.54 ± 3.06 μM	3 ± 1.03 μM	6.51 ± 2.79 μM	3.33 ± 3.72 μM
**Increase in cytotoxicity**	-	1.5-fold	-	1-fold
		**VINBLASTINE**		
**24 h**	NS	NS	NS	NS
**Increase in cytotoxicity**	-	-		
**48 h**	NS	0.01 ± 4.09 nM	NS	0.01 ± 1.88 nM
**Increase in cytotoxicity**		Significant IC_{50}		Significant IC_{50}
**72 h**	0.001 ± 2.04 nM	0.0001 ± 1.54 nM	0.02 ± 4.02 nM	0.001 ± 2.42 nM
**Increase in cytotoxicity**	-	9-fold		19-fold
		**VINCRISTINE**		
**24 h**	NS	NS	NS	NS
**Increase in cytotoxicity**	-	-		
**48 h**	NS	0.009 ± 3.56 nM	NS	0.001 ± 1.17 nM
**Increase in cytotoxicity**	-	Significant IC_{50}		Significant IC_{50}
**72 h**	0.003 ± 1.50 nM	0.0001 ± 8.51 nM	0.05 ± 3.39 nM	0.0001 ± 1.58 nM
**Increase in cytotoxicity**	-	29-fold		499-fold

Not significant (NS)

Table S2. IC_{50} values of drugs and drug combinations tested in Huh-7D cell lines over 72 h.

	**IC_{50}(drug) MTT**	**IC_{50} (drug-combination) (MTT)**	**IC_{50} (drug) Trypan blue**	**IC_{50} (drug-combination) Trypan blue**
		**DOXORUBICIN**		
**24 h**	3.79 ± 7.5 μM	2.16 ± 1.3 μM	4.744 ± 4.16 μM	3.338 ± 2.081 μM
**Increase in cytotoxicity**	-	0.75-fold	-	0.42-fold
**48 h**	2.11 ± 1.46 μM	0.58 ± 4.55 μM	2.949 ± 6.11 μM	1.903 ± 1.73 μM
**Increase in cytotoxicity**	-	2.6-fold	-	0.5-fold
**72 h**	1.7 ± 2.1 μM	0.61 ± 2.2 μM	1.631 ± 2.51 μM	0.6389 ± 2.64 μM
**Increase in cytotoxicity**	-	1.7-fold	-	1.6-fold
		**PACLITAXEL**		
**24 h**	NS	NS	NS	NS
**Increase in cytotoxicity**	-	-		
**48h h**	37.04 ± 1.63 nM	5.60 ± 2.88 nM	41.08 ± 1.52 nM	9.945 ± 3.60 nM
**Increase in cytotoxicity**	-	5.6-fold	-	3.13-fold
**72 h**	31.48 ± 7.48 nM	7.09 ± 2.80 nM	32.57 ± 3.21 nM	6.204 ± 2.51 nM
**Increase in cytotoxicity**	-	3.4-fold	-	4.2-fold
		**OXALIPLATIN**		
**24 h**	NS	NS	NS	NS
**Increase in cytotoxicity**	-	-		
**48 h**	17 ± 1.57 μM	1.06 ± 4.3 μM	23.58 ± 4.93 μM	8.49 ± 1.154 μM
**Increase in cytotoxicity**	-	15-fold	-	1.7-fold
**72 h**	15 ± 7.48 μM	0.37 ± 5.86 μM	16.83 ± 2.08 μM	2.135 ± 1.52 μM
**Increase in cytotoxicity**	-	39-fold	-	6.9-fold
		**VINBLASTINE**		
**24 h**	NS	NS	NS	NS
**Increase in cytotoxicity**	-	-	-	-
**48 h**	0.029 ± 3.16 nM	0.003 ± 8.53 nM	0.049 ± 2.51 nM	0.004 ± 7.63 nM
**Increase in cytotoxicity**	-	8.6-fold	-	9-fold
**72 h**	0.003 ± 6.30 nM	0.0003 ± 5.39 nM	0.004 ± 3.05 nM	0.0003 ± 1.73 nM
**Increase in cytotoxicity**	-	9-fold	-	12-fold
		**VINCRISTINE**		
**24 h**	NS	NS	NS	NS
**Increase in cytotoxicity**	-	-	-	-
**48 h**	0.03 ± 2.92 nM	0.001 ± 1.31 nM	0.012 ± 2.08 nM	0.006 ± 1.53 nM
**Increase in cytotoxicity**	-	29-fold	-	1-fold
**72 h**	0.002 ± 6.95 nM	0.0001 ± 9.53 nM	0.003 ± 3.60 nM	0.0002 ± 4.16 nM
**Increase in cytotoxicity**	-	19-fold	-	14-fold

Table S3. IC_{50} values of drugs and drug combinations tested in SK-hep-1 cell lines over 72 h.

	IC_{50} (drug) MTT	IC_{50} (drug-combination) (MTT)	IC_{50} (drug) Trypan blue	IC_{50} (drug-combination) Trypan blue
		**DOXORUBICIN**		
**24 h**	NS<br>-	NS<br>-	NS	NS
**48 h**	NS	2.38 ± 2.86 μM<br>Significant IC_{50}	NS	2.55 ± 3.88 μM<br>Significant IC_{50}
**72 h**	3 ± 4.34 μM<br>-	2.4 ± 1.21 μM<br>0.25-fold	4.034 ± 5.12 μM<br>-	2.14 ± 3.85 μM<br>0.88-fold
		**PACLITAXEL**		
**24 h**	NS	NS	NS	NS
**48 h**	NS	NS	NS	NS
**72 h**	NS	NS	NS	NS
		**OXALIPLATIN**		
**24 h**	NS	NS	NS	NS
**48 h**	NS	NS	NS	NS
**72 h**	NS	NS	NS	NS
		**VINBLASTINE**		
**24 h**	NS	NS	NS	NS
**48 h**	NS	NS	NS	NS
**72 h**	NS	NS	NS	NS
		**VINCRISTINE**		
**24 h**	NS	NS	NS	NS
**48 h**	NS	NS	NS	NS
**72 h**	NS	NS	NS	NS

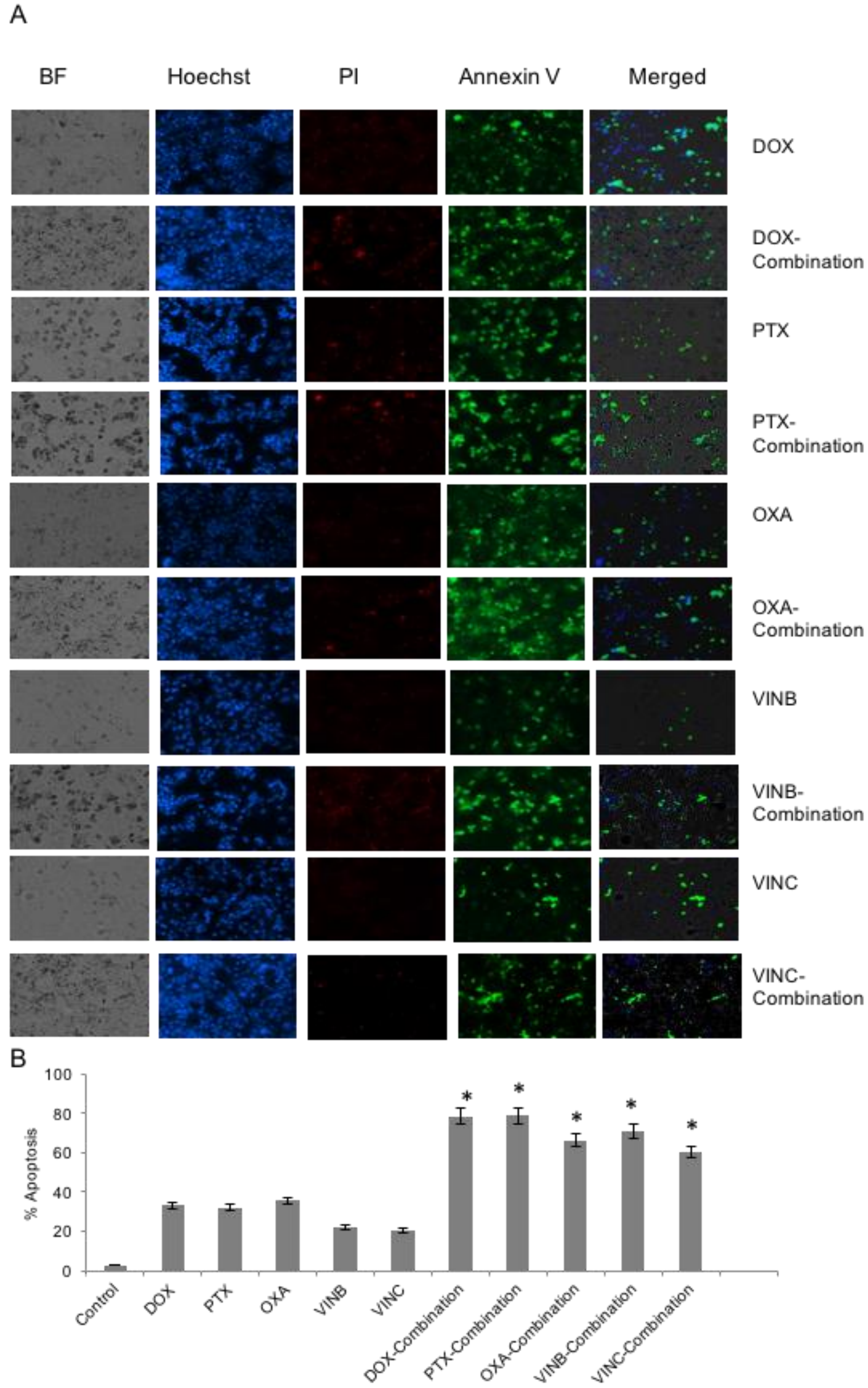

Figure S1. Apoptosis detection cells with Annexin V staining probe. Huh-7D cells treated with single drugs and in combination with HNP-c (hybrid nanoparticle with cyctochrome C conjugated) demonstrated as **A**) images and **B**) quantitatively (n = 3, ±SD). * denotes significance compared to single drug treatment ($p < 0.05$).

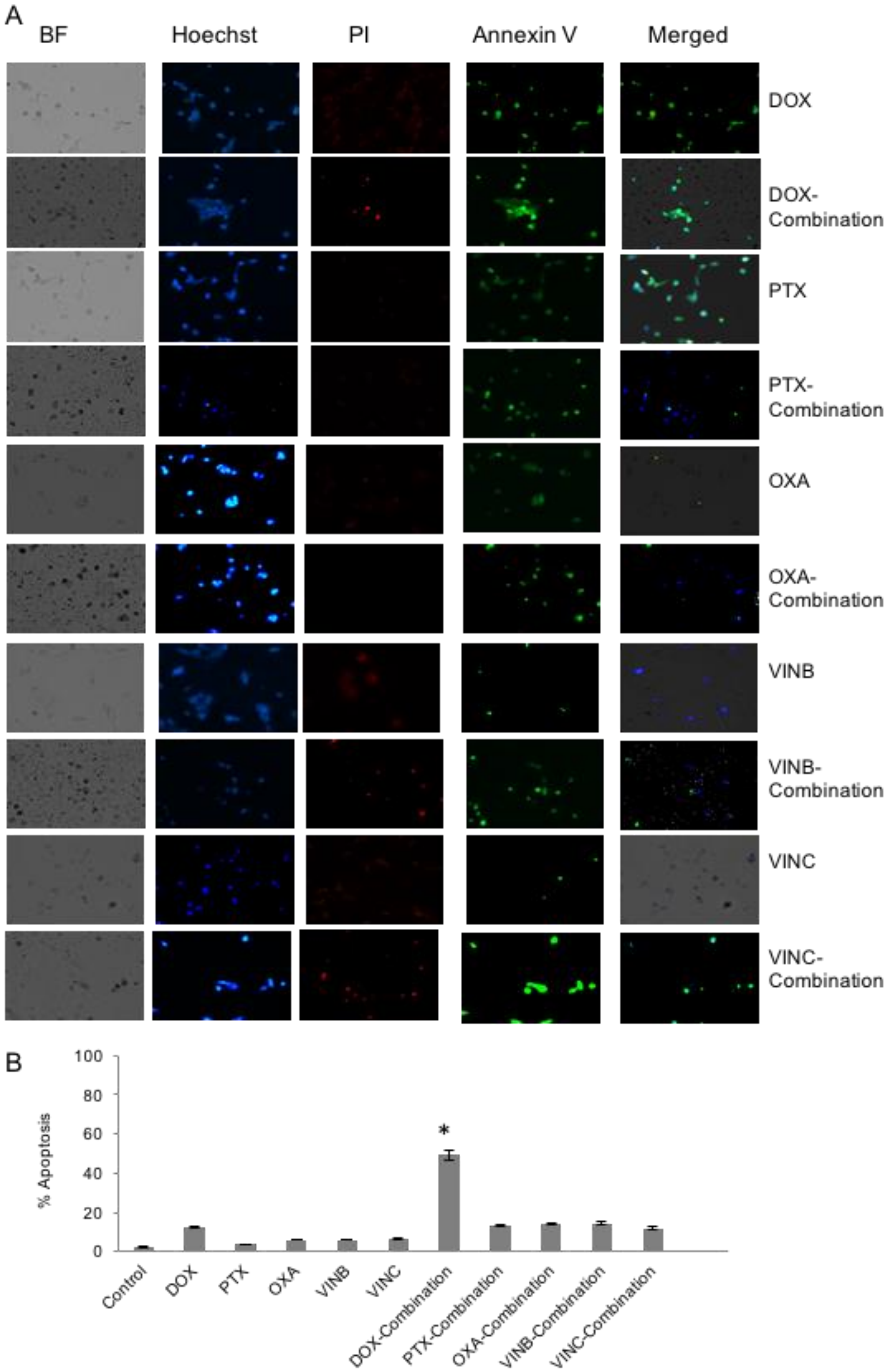

Figure S2. Apoptosis detection cells with Annexin V staining probe. SK-hep-1 cells treated with single drugs and in combination with HNP-c (hybrid nanoparticle with cyctochrome C conjugated) demonstrated as **A**) images and **B**) quantitatively (n = 3, ±SD). * denotes significance compared to single drug treatment ($p < 0.05$).
